# Precipitates in Additively Manufactured Inconel 625 Superalloy

**DOI:** 10.3390/ma12071144

**Published:** 2019-04-08

**Authors:** Beata Dubiel, Jan Sieniawski

**Affiliations:** 1 Faculty of Metals Engineering and Industrial Computer Science, AGH University of Science and Technology, Al. Mickiewicza 30, 30-059 Kraków, Poland; 2 Faculty of Mechanical Engineering and Aeronautics, Rzeszów University of Technology, 2 Wincentego Pola St., 35-959 Rzeszów, Poland; jansien@prz.edu.pl

**Keywords:** Inconel 625, additive manufacturing, laser powder-bed fusion, laser directed energy deposition, microstructure, precipitates, transmission electron microscopy

## Abstract

Laser-based additive manufacturing processes are increasingly used for fabricating components made of nickel-based superalloys. The microstructure development, and in particular the precipitation of secondary phases, is of great importance for the properties of additively manufactured nickel-based superalloys. This paper summarizes the literature data on the microstructure of Inconel 625 superalloy manufactured using laser-based powder-bed fusion and directed energy deposition processes, with particular emphasis on the phase identification of precipitates. The microstructure of Inconel 625 manufactured by laser-based directed energy deposition in as-built condition is investigated by means of light microscopy and transmission electron microscopy. Phase analysis of precipitates is performed by the combination of selected area electron diffraction and microanalysis of chemical composition. Precipitates present in the interdendritic areas of as-built Inconel 625 are identified as MC and M_23_C_6_ carbides as well as the Laves phase.

## 1. Introduction

Inconel 625 is a nickel-based superalloy widely used for both high and low-temperature applications requiring the combination of high strength, corrosion resistance, good formability, and weldability. INCONEL^®^ is the trademark of the Special Metals Corporation group [1] and therefore other companies use different trade names, like Haynes^®^ 625 [2], VDM^®^ Alloy 625 Nicrofer 6020 hMo [3], ATI 625^TM^ [4], Alloy 625 [5], Altemp 625 [6], IN625 [7], or Inconel 625-0402 [8]. In this paper, as in other publications devoted to this alloy [9,10,11,12,13,14,15,16,17], the name Inconel 625 is used. Nominal chemical composition of INCONEL^®^ 625 provided by Special Metals Corporation is the following (in wt.%): Ni (balance, 58.00 min), Cr (20.00–23.00), Mo (8.00–10.00), Nb plus Ta (3.15–4.15), Fe (≤5.00), Ti (≤0.40), Al (≤0.40), Co (≤1.0), C (≤0.10), Si, Mn (each ≤ 0.50), S, P (each ≤ 0.015) [1]. In the conventional cast or wrought form, the mechanical strength of this alloy is derived primarily from solid solution strengthening of the γ phase matrix by Cr, Mo, and Nb. Although Inconel 625 is classified as solid solution strengthened superalloy, its additional strengthening may be derived by precipitation of carbides and/or intermetallic phases. 

For high-temperature applications up to 800 °C Inconel 625 undergoes solution annealing and in such condition it exhibits good creep and thermal-fatigue properties as well as hot gas corrosion resistance, particularly chlorination. Therefore Inconel 625 is extensively used for aerospace and chemical industry applications [2,3]. 

The excellent resistance to different forms of corrosion in aggressive environments also makes it often used at low-temperature in the chemical process industry, sea water and power plant applications. For these purposes, the heat treatment of Inconel 625 comprises soft annealing [2,3]. 

Production of Inconel 625 components with complex shapes is difficult due to its high hardness, poor machinability and low thermal conductivity [18,19]. The use of alternative fabrication route by additive manufacturing (AM) makes it possible to overcome the limitations of producing complex shapes. Therefore, already at the beginning of its development, AM technology was adopted for fabricating components made of Inconel 625 [20]. Several AM processes using laser, electron beam, or plasma arc as an energy source for selective melting of Inconel 625 powder were developed [9,20,21,22]. The two categories of AM processes pertain to Inconel 625, namely powder-bed fusion (PBF) and directed energy deposition (DED) [23]. PBF is the process in which thermal energy selectively fuses chosen areas of a powder bed. In turn, in DED process energy source is focused to melt materials being deposited. Laser-based AM processes are more widespread and therefore investigation of the microstructure of Inconel 625 produced by those methods is of great research interest. 

### 1.1. Laser-Based Powder Bed Fusion Processes

PBF processes, in which laser beam energy is used, are marked with L-PBF acronym. Commercially available L-PBF systems have different trade names, such as Selective Laser Sintering (SLS^®^, which denotes the process and machines from 3D Systems Corporation [24]), Selective Laser Melting (SLM, patented by Fraunhofer Institute ILT in Aachen [25]) Direct Metal Laser Sintering (DMLS^®^, which denotes metal-based laser sintering systems from EOS GmbH [24]), and LaserCUSING^®^ (process patented by Concept Laser [26]). Although the powder bonding processes are considered as sintering or melting, according to ASTM Standard Terminology for Additive Manufacturing Technologies [24] in laser sintering AM machines partial or full melting of fused powder particles occurs. This allows to treat “sintering” as a misnomer term and put a clear distinction between L-PBF processes and conventional metal powder sintering with traditional powder metallurgy methods.

In the L-PBF process, the laser beam is directed to the powder bed and is scanned along a path lying in a cross section of the layer defined in 3D computer model, fusing the powder particles. The powder bed, together with the part being built, is gradually lowered and covered with successive layers of powder, in which the laser beam fuses it along the predetermined path and further layers are incrementally added to the built part. The thickness of the powder layer is greater than the thickness of the layer fused in the single path. However, the penetration depth of laser beam is usually greater than the thickness of three fused layers at a depth of the part, which ensures better bonding of the deposit [27,28,29]. 

### 1.2. Laser-Based Directed Energy Deposition Processes

DED processes using a laser heat source to melt a stream of powder feedstock are marked with the L-DED acronym. The literature terms concerning L-DED are diverse, among others: direct metal deposition (DMD^TM^), laser engineered net shaping (LENS^TM^), laser metal deposition (LMD^TM^), directed light fabrication (DLF^TM^), 3D laser cladding [10,27], or laser solid forming (LSF) [15]. This process is usually used for deposition of metals in powder (or wire) form on the substrate material. In L-DED the metal powder is fed through a nozzle with an inert gas to the focal point of the laser beam or to the position of the movable molten pool. A significant difference between L-PBF and L-DED is that in L-DED the powder fully melts, so the microstructure is formed by rapid crystallization from the liquid phase. Typical applications of L-DED include the repair and maintenance of structural parts [27].

### 1.3. Microstructure of Inconel 625 Manufactured by Laser-Based Additive Manufacturing Processes

In AM processing of superalloys cooling rates during solidification are in the range of 10^3^–10^6^ °C⋅s^−1^, much higher than in traditional cast and wrought processes, thus the microstructure and mechanical properties are quite different than those of their conventionally produced counterparts [9]. Due to the rapid melting followed by the rapid cooling in L-PBF and L-DED processes, Inconel 625 in the post-built condition exhibits fine dendritic microstructure. 

Additively manufactured Inconel 625 routinely undergoes stress-relief annealing at a temperature of 870 °C for 1 h, so the dendritic microstructure, and in consequence microsegregation, remain [9]. Optionally, the solution annealing at a higher temperature up to 1150 °C can be performed [30,31]. The inhomogeneity of the microstructure influences the mechanical properties, but its effect on material performance is not yet fully understood. 

Characterization of the Inconel 625 microstructure in the post-built and stress-relieved conditions was the subject of several studies [9,10,11,12,13,14,15,16,17,20,28,29,30,31,32,33]. However, no work has demonstrated the comprehensive description of the precipitates present in as-built condition or after subsequent heat treatment of Inconel 625 manufactured by laser-based additive manufacturing processes.

Precipitates in other additively manufactured materials were extensively studied in previously published reports, e.g., in precipitate hardenable 17-4 stainless steel [34,35], maraging steel [36], scandium-modified aluminium alloy [37], or Inconel 718 [38]. Meanwhile, literature data on the precipitates in L-PBF and L-DED fabricated Inconel 625 is not very extensive. In the following subsections, an overview of the current state of knowledge on this subject is presented.

#### 1.3.1. Precipitates in Inconel 625 Manufactured by L-PBF Process

The literature data concerning the microstructure of Inconel 625 manufactured by L-PBF is slightly more extensive than in the case of DED processing. Scientific papers published in recent years reflect the increased interest in the problems related to the fabrication, microstructure and properties of Inconel 625 manufactured by L-PBF processes [9,11,12,13,14,15,16,17,28,29,30,31,32,33]. Particularly, phase transformations in Inconel 625 produced by L-PBF are attracting the growing interest of researchers. In several papers, results of the investigation of precipitates in as-built, stress-relief and solution annealing Inconel 625 have been reported. 

In the as-built Inconel 625 Amato et al. [28] observed precipitates rich in Nb, located along the melt pool boundaries, which were identified as the γ″ phase. The provided results of transmission electron microscopy (TEM) imaging, microanalysis by energy-dispersive X-ray spectroscopy (EDS) as well as phase identification by selected area electron diffraction (SAED) and X-ray diffraction (XRD) are not convincing, and it cannot be excluded that the observed precipitates are the δ phase. In turn, Zhang et al. [29], using EDS elemental maps acquired in a scanning electron microscope (SEM), revealed significant segregation of Ni and Cr to dendrite cores as well as enrichment of the interdendritic regions in Nb and Mo in the as-built Inconel 625. Their research did not involve the investigation of secondary phases. Meanwhile, based on SEM imaging and XRD results Lass et al. [9] postulated that in the as-built condition the microstructure consists of the single-phase γ solid solution. The nano-sized precipitates in the γ matrix of the as-built samples, exhibiting bright contrast in scanning-transmission electron microscopy (STEM) images acquired in the High Angle Annular Dark Field (HAADF) mode, were observed by Li et al. [30]. Phase analysis of these precipitates was not performed, however, EDS microanalysis showed that they are enriched in Nb and Mo. Likewise, TEM microstructural analysis performed by Keller at al. [31] has not allowed to uniquely identify precipitates in the as-built Inconel 625. Later Marchese et al. [14] observed nano-sized precipitates rich in Nb, which may be Nb-rich MC carbides. Moreover, irregular particles in the interdendritic zones were interpreted as segregations of heavy metals such as Nb and Mo.

The effect of stress-relief annealing on the microstructure of Inconel 625 was investigated by several research groups [9,29,31,32]. SEM microstructural analysis performed by Lass et al. [9] revealed precipitates with plate-like morphology located in the interdendritic regions. EDS microanalysis showed that these precipitates are enriched in Nb and Mo. XRD and SAED investigation allowed to identify them as the δ phase precipitates. Further studies performed by TEM allowed them to confirm the existence of δ and γ″ phases [32]. After annealing at a lower temperature of 800 °C for 1 h, Nb- and Mo-rich precipitates were also found. Based on the EDS elemental maps these precipitates were suspected to be MC or M_6_C carbides, rather than the δ phase, due to the poor signal in Ni map [9]. 

Likewise, Keller at al. [31] carried out TEM microstructural analysis of Inconel 625 subjected to stress-relief annealing. Their results of phase analysis using SAED confirmed the presence of MC, M_6_C and M_23_C_6_ carbides. Additionally, they performed phase analysis by means of synchrotron X-ray diffraction at the ultra-small angle X-ray scattering (USAXS). X-ray diffraction patterns revealed the presence of M_6_C carbides in the specimen annealed at 870 °C. 

In solution annealed Inconel 625 secondary phases were detected by several research groups. Li et al. [30], based on the morphology of precipitates observed by means of STEM-HAADF, as well as EDS microanalysis of chemical composition, suspected that possibly two kinds of particles, round Cr oxides, and square Cr-rich precipitates are present. Keller et al. [31] have proven using X-ray synchrotron diffraction that after annealing at temperature 1150 °C the M_6_C carbides and the Laves phase are the dominant precipitates. Also, Marchese et al. [10] investigated the microstructure of Inconel 625 after solution annealing and observed the presence of coarse primary as well as small secondary Nb- and Ti-rich MC carbides.

Furthermore, Keller et al. [31] performed thermodynamic calculations of the driving forces for nucleation precipitates at the temperature of 870 °C and 1150 °C, corresponding to stress-relief and solution annealing of L-PBF Inconel 625. The Gibbs free energy was calculated for several possible phases which are likely to precipitate from the γ matrix, namely MC, M_2_C, μ, M_6_C, α, δ, P, Laves phase, σ, γ″, and M_23_C_6_. Comparison of the driving force values allowed to propose the sequence of phases expected most likely to nucleate, starting from Nb-rich MC and M_2_C, Mo-rich M_6_C, or Mo-rich solid solution with body centred cubic structure, and then Cr-rich phases σ, P, Laves, or M_23_C_6_. The precipitation sequence predicted by thermodynamic calculation performed in [9] was not confirmed yet by the results of microstructural studies.

#### 1.3.2. Precipitates in Inconel 625 Manufactured by L-DED Processes

The microstructure of Inconel 625 achieved by L-DED was investigated by Dinda et al. [20]. The studies were devoted mostly to the characterization of the dendritic microstructure in as-built condition and after annealing at the temperature range 700–1200 °C for 1 h. It was determined that the dendritic microstructure remains stable up to the temperature of 1000 °C. The light microscopy (LM) and XRD investigation did not allow to detect the presence of precipitates within the γ matrix. The observed differences in the microhardness as well as in the lattice parameter of the γ phase matrix between the as-built and annealed conditions were suspected to be related with precipitation and dissolution of either γ″ or δ phases.

In turn, Marchese et al. [10] examined the microstructure of as-built Inconel 625 manufactured by L-DED using SEM and EDS. Their observations indicate the possibility of the presence of the Laves phase rich in Nb, Mo, and Si, Nb-rich carbides as well as ellipsoidal precipitates around 100 nm in size, identified as the γ″ phase. Similarly, Hu et al. [15] postulated that precipitates rich in Nb, Mo, and Si, detected by SEM and EDS in the interdendritic regions of L-DED Inconel 625, are the Laves phase particles. However, SEM imaging and EDS microanalysis of chemical composition allow only to postulate which phases can be present. 

Based on a critical review of the literature, it is worth emphasizing that it is still not well established whether and which precipitates are present in the as-built Inconel 625. For example, in the review paper [39] it was stated that the microstructure of the as-built L-DED Inconel 625 consists exclusively of the γ phase.

Moreover, from the literature review, it is apparent that precipitates in as-built L-DED Inconel 625 were not examined using TEM. On the other hand, the results of TEM microstructural investigation of Inconel 625 alloy in a similar state to L-DED are available, namely in the form of laser cladded coating. Verdi et al. [33] examined precipitates in laser cladded Inconel 625 using TEM and high-resolution electron microscopy (HRTEM) imaging in combination with SAED and EDX analysis. In as-deposited material, precipitates enriched in Nb and Mo were identified as M_6_C and M_2_C carbides. Moreover, the presence of the γ″ phase particles was confirmed. 

Likewise as in the case of L-PBF Inconel 625, the identification of precipitates in L-DED manufactured superalloy is not straightforward. Due to the similarities in chemical composition and small differences of lattice parameters between various phases, it is still controversial which phases are present. Commonly precipitates are recognized based on their shape and chemical composition, however, these features are not sufficient for phase ana lysis. Moreover, information about the influence of chemical elements on the formation of the different phases in the L-DED Inconel 625 is still limited. Hence, it is important to find the simple method that is suitable for the identification of precipitates. The novelty of this study is the use of selected area electron diffraction patterns solved with the use of simulated spot diffraction patterns and EDS microanalysis of chemical composition for identification of precipitates in the L-DED Inconel 625 in as-built condition. According to our knowledge, it is the first study which shows that the identification of phases by this approach is unambiguous and allows to eliminate inaccuracies resulting from the recognition of particles in the as-built L-DED Inconel 625 based on their morphology and chemical composition.

## 2. Materials and Methods

The sample of Inconel 625 produced by L-DED was delivered by Infinitech 3D (Radom, Poland) [40] in the form of a square grid sample with the wall thickness of 17 mm in as-built condition. The Inconel 626 powder was delivered by Höganäs, Ath, Belgium. The manufacturer used Powder Fed Laser Metal Deposition Technology of RPM Innovations Inc. (Rapid City, SD, USA) and RPMI 557 machine. Process parameters elaborated at Infinitech 3D are confidential and therefore are not specified. The chemical composition of the alloy is given in Table 1. 

Microstructural characterization was performed using light microscopy (LM) and TEM. The polished and etched specimen prepared from the longitudinal section (x-z plane) of the sample was examined using Axio Imager M1m light microscope (ZEISS, Jena, Germany). Thin foils for TEM investigation were prepared from the cross section (x-y plane) of the sample using electropolishing. TEM imaging and microanalysis of the chemical composition were performed using JEM-2010 ARP microscope (JEOL Ltd., Tokyo, Japan) equipped with INCA EDS microanalysis system (Oxford Instruments, High Wycombe, UK). SAED patterns were analysed with the use of crystallographic data available in AtomWork software (NIMS, Tsukuba, Japan) [41] and JEMS v4.4230 software (Pierre Stadelmann, JEMS-SAAS, Saas-Fee, Switzerland).

## 3. Results and Discussion

### 3.1. Microstructure of As-Built L-DED Inconel 625

**Figure 1:**
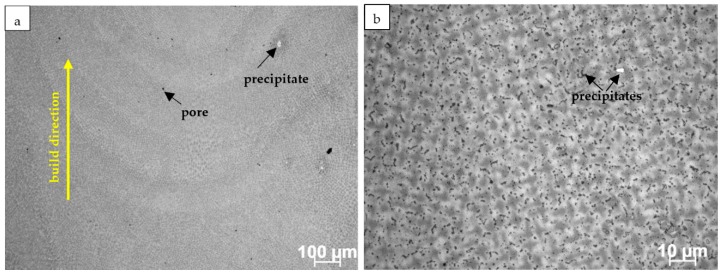
Light microscopy images of the L-DED Inconel 625 microstructure in as-built condition: (**a**) deposited layers with dendritic structure, exemplary pore and precipitate are marked by arrows and (**b**) larger magnification image showing the high density of precipitates, mostly located in interdendritic areas.

Figure 1a,b shows the light microscopy images of the L-DED Inconel 625 in the longitudinal section. In Figure 1a, layers of the deposited material exhibiting dendritic microstructure are visible. In Figure 1b, at larger magnification, dendrites with short secondary arms, etched in a darker colour than interdendritic areas, are arranged in different directions in individual grains, which reflect changes in the main direction of heat flow during crystallization. Pores and precipitates are also observed. 

Pores with oval and irregular shapes are randomly distributed in the observed area of Figure 1a. Generally, it can be assumed that porosity is caused by the entrapping of the carrier gas during repeated re-melting in the multi-layer deposit. However, the pore generation depends on a large number of L-DED process parameters factors. Fujishima et al. [42] examined the influence of major parameters of the L-DED process on the porosity of Inconel 625. Their results indicate that the relationship between laser power, powder feed rate, and carrier gas flow significantly affect porosity. Systematic experiments with various parameters are therefore necessary to elaborate the appropriate process parameters allowing the reduction of porosity. 

Two size ranges of precipitates can be recognized on LM images. White etched precipitates with a size of a few micrometres are observed even at low magnification. Numerous small dark-etched particles are located mostly in the interdendritic areas (Figure 1b). Such observation demonstrates that, due to the dendritic solidification, the pronounced microsegregation of alloying elements occurs, which results in the formation of precipitates.

**Figure 2:**
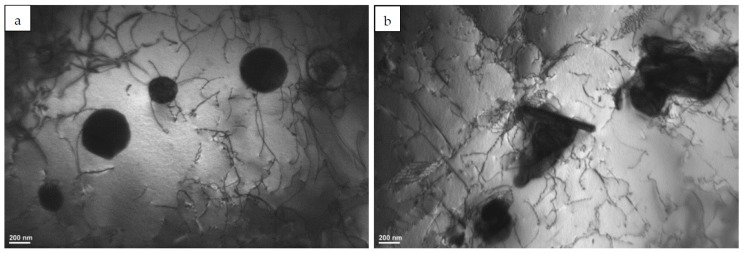
Transmission electron microscopy (TEM) images of precipitates in the as-built L-DED Inconel 625 with: (**a**) globular and spherical morphology, as well as (**b**) merged particles with irregular and/or plate-like morphology.

TEM observations of the microstructure revealed numerous precipitates of various shapes in the interdendritic areas. Figure 2a,b shows examples of precipitates with globular and spherical morphology, as well as merged particles composed of several parts with irregular and/or plate-like shapes. Due to the small size from several tens of nanometres up to several hundreds of nanometres, such precipitates could not be identified with X-ray diffraction. Consequently, further investigation for determining the precipitated phases with different morphologies and chemical compositions was carried out using SAED and EDS in TEM. 

### 3.2. Phase Analysis of Precipitates

**Figure 3:**
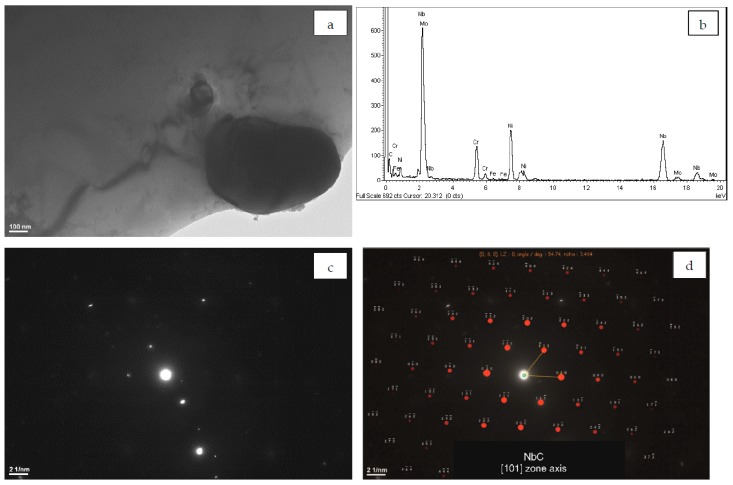
(**a**) TEM image of the MC precipitate; (**b**) the corresponding energy-dispersive X-ray spectroscopy (EDS) spectrum; (**c**) selected area electron diffraction (SAED) pattern; and (**d**) its solution for NbC [101] zone axis.

Figure 3a shows a globular particle and the corresponding EDS spectrum is given in Figure 3b. The spectrum indicates that the particle contains mostly Nb. The peaks of Ni and Cr most probably come from the γ matrix. The smaller peaks of C, Mo, and Fe are also present. The diffraction pattern of the particle is shown in Figure 3c. The solution of the SAED pattern was obtained for the [101]_NbC_ zone axis (Figure 3d). This is in accordance with results of the previous studies on L-DED processed Inconel 625 [10,14]. Due to the pronounced segregation of Nb in the interdendritic regions, MC carbides rich in Nb may precipitate as secondary phases under the influence of thermal cycles during the application of successive layers of material in the L-DED process. Observation of the Nb-rich MC carbides is also consistent with the results of the thermodynamic calculation of the driving force for nucleation of secondary phases in the interdendritic regions performed by Keller et al. [31]. They predict that Nb-rich MC carbides nucleate first from the γ matrix supersaturated with Nb. Another possibility is that MC particles precipitate directly from the liquid phase. According to Dupont et al. [43,44] in Nb-bearing superalloys Nb segregates strongly during solidification to the liquid phase. The Nb-rich MC carbides and the Laves phase precipitates can be formed in eutectic reactions:

L → γ + NbC and L → γ + Laves.

**Figure 4:**
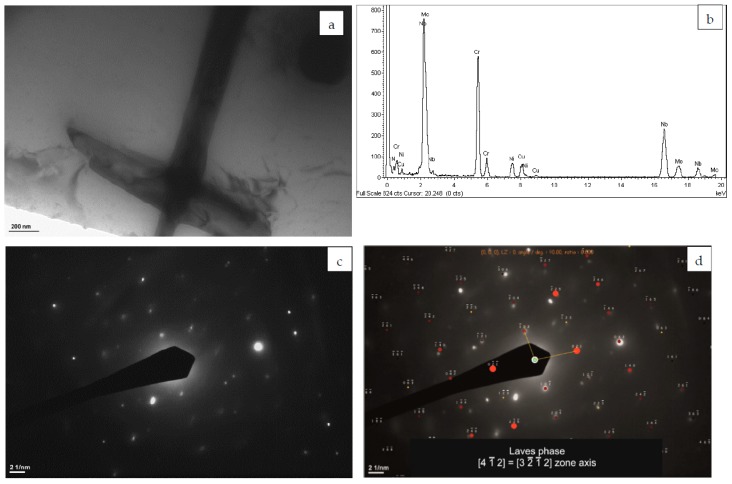
(**a**) TEM image of the Laves phase precipitate; (**b**) the corresponding EDS spectrum; (**c**) SAED pattern; and (**d**) its solution for the Laves phase [4 $\bar{1}$ 2] zone axis.

Results of the TEM study also confirm the presence of the Laves phase. Figure 4a shows an example of the precipitate in the form of merged plate-like particles, which was identified by SAED as the Laves phase (Figure 4c,d). EDS spectrum reveals that the Laves phase is enriched in Nb, Cr, and Mo (Figure 4b). The enrichment of the Laves phase in Cr can be somewhat surprising because it was mainly reported as Nb- and Mo-rich. However, it is in line with the results of Keller et al. [31], who reported the possibility of precipitation of the Cr-rich Laves phase in L-PBF Inconel 625. Precipitates of the Laves phase were also identified by Marchese at al. [10] and Hu et al. [15] in the interdendritic areas of L-DED Inconel 625. Moreover, the Laves phase was present in L-PBF Inconel 625 [31] as well as in the weld overlay [45] and the cold metal transfer clad layers [46]. There is a general agreement that the precipitation of the Laves phase is related to the strong segregation of Nb and Mo to the interdendritic areas. The result of TEM and EDS investigation shows that in as-built L-DED Inconel 625 precipitation of the Nb-, Cr-, and Mo-rich Laves phase occurs.

**Figure 5:**
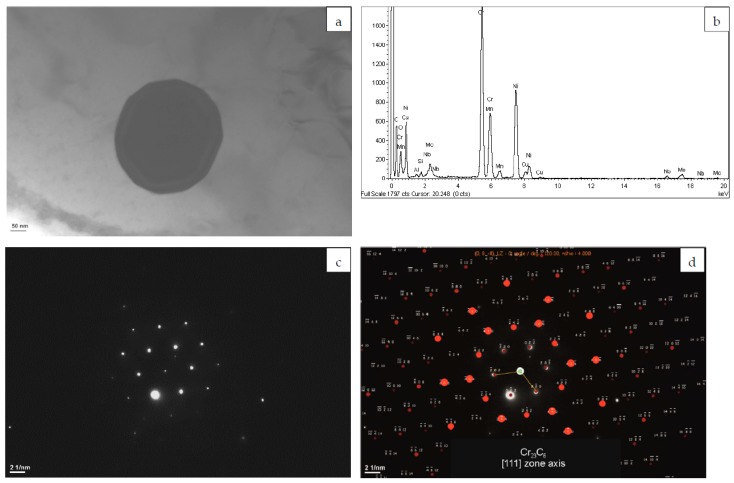
(**a**) TEM image of the M_23_C_6_ precipitate; (**b**) the corresponding EDS spectrum; (**c**) SAED pattern; and (**d**) its solution for Cr_23_C_6_ [111] zone axis.

Another phase rich in Cr, identified in as-built L-DED Inconel 625, is the M_23_C_6_ carbide. Figure 5 shows the TEM image and EDS spectrum of the globular particle enriched in Cr, as well as SAED pattern and its solution. M_23_C_6_ carbides are present in conventional Inconel 625 [47], but in its laser-based additively manufactured form they are rarely observed. Precipitation of Cr-rich M_23_C_6_ carbides in interdendritic areas of L-PBF Inconel 625 subjected to stress-relief annealing was confirmed by Keller et al. [31]. However, based on the results of the thermodynamic calculation, they postulated that the Cr-rich σ, P, and Laves phases are expected to precipitate prior to the M_23_C_6_ [31]. The obtained results confirm that the local variations in chemical composition of the L-DED processed material may favour precipitation of Cr-rich M_23_C_6_ carbides in as-built Inconel 625.

The different phases identified by SAED in TEM confirm that Inconel 625 additively manufactured by L-DED process exhibits a tendency for precipitation of carbides and intermetallic phases even in the as-built condition. 

Further studies would be needed in order to determine the thermal stability of precipitates and thus the possibilities of homogenization of the as-built microstructure leading to the dissolution of the precipitated phases. 

## 4. Conclusions

Inconel 625 superalloy produced by laser-based additive manufacturing processes shows high susceptibility toward precipitation of carbides and intermetallic phases. In the as-built L-DED condition Inconel 625 exhibits very fine dendritic microstructure. LM and TEM investigation revealed the presence of numerous precipitates in interdendritic areas. TEM microstructural investigation combined with EDS microanalysis highlighted the existence of precipitates with diverse morphology and chemical composition. Analysis of SAED patterns allowed to confirm the presence of Nb-rich MC carbides, the Nb-, Cr-, and Mo-rich Laves phase precipitates, as well as M_23_C_6_ carbides, enriched mainly in Cr. 

## Figures and Tables

**Table 1:** Chemical composition of the Inconel 625 sample produced by directed energy deposition using a laser (L-DED).

Chemical Element (wt.%)												
Ni	Cr	Mo	Nb	Fe	Ti	Al	Co	Si	Mn	C	N	O
≥58.00	20.00–23.00	8.00–10.00	3.15–3.85	≤1.50	≤0.10	≤0.10	0	0.30–0.50	0.20–0.50	≤0.03	≤0.14	≤0.07
